# Switchable Polyacrylic Acid Polyelectrolyte Brushes for Surface Plasmon Resonance Applications

**DOI:** 10.3390/s23094283

**Published:** 2023-04-26

**Authors:** Qais M. Al-Bataineh, Ahmad D. Telfah, Victoria Shpacovitch, Carlos J. Tavares, Roland Hergenröder

**Affiliations:** 1 Leibniz Institut für Analytische Wissenschaften-ISAS-e.V., Bunsen-Kirchhoff-Straße 11, 44139 Dortmund, Germany; 2 Experimental Physics, TU Dortmund University, 44227 Dortmund, Germany; 3 Nanotechnology Center, The University of Jordan, Amman 11942, Jordan; 4 Centre of Physics of Minho and Porto Universities, University of Minho, 4804-533 Guimarães, Portugal

**Keywords:** imaging wide-field SPR microscopy sensor, polyacrylic acid (PAA), polyelectrolyte brushes (PEBs)

## Abstract

Imaging wide-field surface plasmon resonance (SPR) microscopy sensors based on polyacrylic acid polyelectrolyte brushes (PAA PEBs) were designed to enhance the sensitivity of nano-object detection. The switching behavior of the PAA PEBs against changes in the pH values was investigated by analyzing the chemical, morphological, optical, and electrical properties. At pH ~1, the brushes collapse on the surface with the dominance of carboxylic groups (COOH). Upon the increase in the pH to nine, the switching process completes, and the brushes swell from dissociating most of the COOH groups and converting them into ${\mathrm{COO}}^{-}$ groups. The domination of the negatively charged ${\mathrm{COO}}^{-}$ groups increases the electrostatic repulsion in the polymer chains and stretches the brushes. The sensitivity of the SPR sensing device was investigated using a theoretical approach, as well as experimental measurements. The signal-to-noise ratio for a Au layer increases from six to eighteen after coating with PAA PEBs. In addition, the linewidth of the recorded image decreases from six pixels to five pixels by using the Au-PAA layers, which results from the enhanced spatial resolution of the recorded images. Coating a Au-layer with PAA PEBs enhances the sensitivity of the SPR sensing device, and improves the spatial resolution of the recorded image.

## 1. Introduction

Polyelectrolyte brushes (PEBs) are charged poly acids or bases grafted to the substrate surface which undergo reversible protonation or deprotonation upon shifting the pH value across the acidity of the brush [1,2]. This shift allows for the binding of the PEBs with their target molecules [3], and consequently has various applications in biosensors and bioelectronic devices [4,5].

The physical binding between the polyelectrolyte brushes with the macromolecules remains an open question. For instance, several researchers have stated that the proteins bind to the PEBs even when they have the same net charge [6], which can be attributed to the interactions with the local patches on the protein surface that have the opposite charge to the PEBs [7,8], or that the local environment inside PEBs reverses the protein charge [9]. Using different chemical modifications, the PEBs can coat surfaces of different materials, such as metals, metal oxides, silicon, and silica [10].

Polyacrylic acid (PAA) has gained attention due to the existence of a carboxylic group on each repeating unit that changes its charges, structures, and hydrophilicity due to the variation in pH [11,12]. Increasing the pH value of the PAA PEBs leads to the deprotonation of COOH and converting it into the ${\mathrm{COO}}^{-}$ of the carboxylic group. The dominance of the negatively charged ${\mathrm{COO}}^{-}$ groups increases the electrostatic repulsion in the polymer chains, consequently stretching the brushes and converting the PAA into a hydrophilic behavior. Moreover, the reduction in the value of the PAA PEBs leads to the re-protonation of ${\mathrm{COO}}^{-}$ groups, and the PEBs return to their initial state [13].

Recently, imaging wide-field SPR microscopy sensors (WF-SPRM) have gained substantial attention in the real-time detection of viruses and virus-like particles due to their capability to measure low particle concentrations with a high sensitivity [14,15,16]. In addition, the WF-SPRM of nano-objects is a spatio-temporal detector based on SPR phenomena for imaging the nano-objects in solution and gas media due to the interaction between nano-objects with the evanescent electrical field near the sensor surface [17,18,19,20,21,22].

Various studies have been conducted to enhance the sensitivity and selectivity of the SPR sensors [23]. G. del Castillo and colleagues [3] investigated the binding of enzymes to the polyelectrolyte brushes compared to self-assembled monolayers. They concluded that the application of polyelectrolyte brushes enhances enzyme binding. Y. Wang et al. [24] found that the polyacrylic acid polyelectrolyte brushes demonstrated a higher selectivity to protein loading than bare gold surfaces.

This work describes the design of the WF-SPRM based on PAA PEBs deposited on a Au layer, which improves the sensitivity of the SPR sensing device. Fourier transform infrared (FTIR) and nuclear magnetic resonance (NMR) spectroscopies were employed to investigate the transformation of the carboxylic acid (COOH) group into the carboxylate (${\mathrm{COO}}^{-}$) group of the PAA PEBs with increasing pH values due to added NaOH. Atomic force microscopy (AFM) was used to monitor the transformation of the carboxylic acid (COOH) group into the carboxylate (${\mathrm{COO}}^{-}$) group, which changes the PAA PEBs from a collapsed to a stretched form. The effect of switching the behavior on the optical constants and electrical conductivity was investigated. Finally, PAA PEBs were chosen for the SPR sensing device due to their high refractive index. Theoretical calculations, in addition to experimental measurements of WF-SPRM, were used to investigate the sensitivity of the SPR sensing device.

## 2. Materials and Methods

Polyacrylic acid (${\left({\mathrm{CH}}_{2}-{\mathrm{CHCO}}_{2}H\right)}_{n}$, 1800 g/mol), N-hydroxy succinimide ($C_{4}H_{5}{\mathrm{NO}}_{3}$, 115.09 g/mol), cysteamine hydrochloride ($C_{2}H_{7}\mathrm{NS}$, 113.61 g/mol), toluene (C₆H₅CH₃, 92.14 g/mol), hydrochloric acid (HCl, 36.458 g/mol), and sodium hydroxide (NaOH, 39.997 g/mol) were all purchased from Sigma-Aldrich, except for 1-ethyl-3-(3-(dimethylamino) propyl) carbodiimide) hydrochloride ($C_{8}H_{17}N_{3}$, 155.245 g/mol), which was purchased from ThermoFisher. The following sections describe the synthesis and characterization methods in detail.

### 2.1. Synthesis of Thiolated Polyacrylic Acid (PAA-SH)

PAA stock solution was prepared by dissolving 1.000 g polyacrylic acid in 100 mL distilled water using a magnetic stirrer for four hours under ambient conditions. As according to the literature [25], thiolated polyacrylic acid (PAA-SH) was synthesized via carbodiimide amide coupling, in which 0.320 g of N-hydroxy succinimide and 0.533 g of 1-ethyl-3-(3-(dimethylamino) propyl) carbodiimide) hydrochloride were added into 100 mL of PAA stock solution in a 6:1 molar ratio under continuous stirring for 30 min at room temperature. After that, 0.316 g of cysteamine hydrochloride in a 6:1 molar ratio (carboxyl to thiol) was added to the reaction, and stirred continuously for four hours at room temperature. Finally, the PAA-SH PEBs were purified by adding toluene to small amounts of the final solution (100 mL toluene to 10 mL PAA-SH solution). After that, PAA-SH powder was collected using a centrifuge system. Finally, 1 g of PAA-SH was suspended in 100 mL of absolute ethanol for further use. The pH value of the solution was 2.

The pH value of the preparation solvent varied from 1 to 9, which was controlled by adding hydrochloric acid (HCl) or sodium hydroxide (NaOH) to the final solution. Adding HCl leads to a reduction in pH through the delivery of ${\mathrm{Cl}}^{-}$ ions to the solvent preparation. However, adding NaOH leads to an increase in pH via the delivery of ${\mathrm{Na}}^{+}$ ions to the solvent preparation.

### 2.2. Surface Cleaning

Before surface functionalization, indium-tin-oxide (ITO) glass substrates (100 nm ITO on glass) and n-type silicon wafers (0.5 mm-thick) were cleaned by sonicating in acetone and isopropyl alcohol for 30 min under ambient conditions. In addition, the gold substrates were prepared by first coating a glass slide (14 × 75 × 1 mm^3^, SF10 glass, *n* = 1.72) with a 5 nm titanium adhesion layer and, subsequently, with an approximately 41-45 nm gold layer via a magnetron sputtering technique (Innolume, Dortmund, Germany). These gold substrates were further cut and cleaned using piranha solution ($H_{2}{\mathrm{SO}}_{4}/H_{2}O_{2}$, 3:1 *v*/*v*) for 10 min. Afterward, ITO, silicon, and the gold substrates were rinsed with water and dried at ambient conditions.

### 2.3. Brushes by the “Grafting to” Method

PAA PEBs were deposited on the gold, ITO, and silicon substrates as according to the “grafting to” method by modifying the PAA brushes using the thiol group. The PAA-SH PEBs were deposited via the spin coating technique. Afterward, the films dried at 70 °C to vaporize the solvent. ITO substrate was used for optical and electrical characterizations, an n-type silicon wafer was used for X-ray photoelectron spectroscopy experiments, and a gold substrate was used for SPR experiments.

### 2.4. Chemical Structure Characterizations

The chemical structure was investigated using ^1^H NMR measurements (600.13 MHz, Bruker AVANCE III NMR spectrometer) and near-ambient-pressure X-ray photoelectron spectroscopy (NAP-XPS). ^1^H NMR measurements were conducted at 600.13 MHz using a 5 mm high-resolution multi-channel and broadband (5 mm BBO model) equipped with Z-gradient.

### 2.5. The Swelling Behavior Characterizations

The swelling behavior and the dissociation degree were investigated using FTIR (Bruker VERTEX 80/80v Vacuum FTIR Spectrometers) and NMR (Bruker AVANCE III NMR spectrometer). In addition, surface morphology and wettability were studied using an atomic force microscope (SPM SmartSPM™-1000) and water contact angle measurements (WCA) for a water droplet (pH = 7) of size 10 μL taken on three occasions. These measurements were performed at room temperature for PAA-SH PEBs at different pH values (1, 4, 7, and 9, respectively).

### 2.6. Optical and Electrical Properties

The optical transmittance and reflectance spectra were measured using a double-beam UV–Vis spectrophotometer (Hitachi U-3900H) with a total integration sphere. After that, the optical constants (extinction coefficient $\left(k\right)$ and refractive index (n)) were calculated using k=αλ/4π and $n=\left(1+R/1-R\right)+\sqrt{\left(4R/{\left(1-R\right)}^{2}\right)-k^{2}}$, respectively, where α is the absorption coefficient, given by $\alpha =\left(1/d\right)\ln\left(\left(1-R\right)/T\right)$ [26,27], λ is the photon wavelength, and d is the thickness. The n-spectra were fitted to the Cauchy model [28]. A 4-point probe (Microworld Inc., Kuala Lumpur, Malaysia) was used to measure the electrical conductivity.

### 2.7. WF-SPRM Measurements

The SPR instrument employed for measurements with the (PAA PEBs–Au) layer is based on Kretschmann’s scheme of plasmon excitation [29,30]. The PAA-SH PEBs with 20 nm thickness were deposited onto the gold substrate using the spin coating technique. The gold substrate was pressed onto a glass prism using RI matching immersion liquid (n=1.725, Cargill Laboratories, Fort Collins, CO, USA) to avert the air gap. SPR measurements were performed by illuminating the (PAA PEBs–Au) layer through a prism using a laser diode (HL6750MG, Thorlabs GmbH, Bergkirchen, Germany) with a wavelength of λ=685 nm. Images of the gold layer interface were taken using a CMOS video camera DMK 23UP031 (Imaging Source, Bremen, Germany, with a resolution of 5 Mp (megapixel) and a pixel size 2.2 × 2.2 µm) and using a macro-objective (Cannon Compact-Macro Lens EF 50 mm 1:2.5). Details of WF-SPRM are described elsewhere [20,31]. The resonance angle represents the minimum residual reflectivity. Finally, polystyrene nanoparticles (refractive index *n* = 1.59 [32]) with an average size of 200 nm were used to simulate the binding events of the biological nanoparticles.

## 3. Results and Discussion

**Figure 1:**
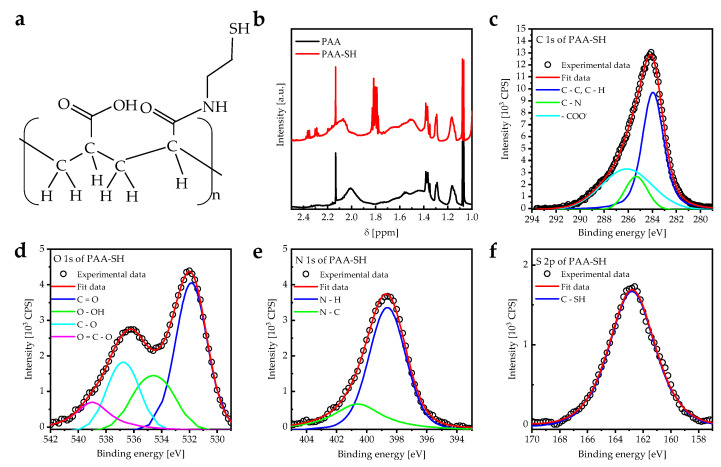
(**a**) Molecular structure of PAA-SH with a carboxyl-to-thiol ratio (1:4). (**b**) ^1^H NMR spectra for PAA and PAA–SH PEBs at pH = 7 measured at room temperature. NMR spectrum of PAA-SH shows the chemical shift of PAA and the -SH group. (**c**) Deconvoluted XPS C 1s spectra of PAA-SH PEBs. (**d**) Deconvoluted XPS O 1s spectra of PAA-SH PEBs. (**e**) Deconvoluted XPS N 1s of PAA-SH PEBs. (**f**) Deconvoluted XPS S 2p spectra of PAA-SH PEBs.

The main challenge in using PAA PEBs as a coating on metal films or other substrates is the lack of a functional group that strongly binds to the surface. Therefore, a fraction of carboxylic groups of PAA were modified with thiol functional groups binding to a gold layer. PAA-SH was synthesized using carbodiimide amide coupling reactions between PAA and cysteamine with specific molar ratios to obtain a modified one in four carboxylic acid groups (Figure 1a) [25]. The ^1^H-NMR spectrum of PAA PEBs shows signals at 1.1, 1.2–1.7, and 2.01 ppm ascribed to ${\mathrm{CH}}_{3}$, ${\mathrm{CH}}_{2}$, and CHCOOX protons, respectively. In addition, the multiplet peaks between 1.76 and 1.85 ppm represent the thiol (–SH) group in the PAA–SH PEBs, which thereby confirms the existence of the –SH group in the PAA matrix (Figure 1b) [33,34]. Performed XPS measurements also confirmed the presence of the thiol group in PAA-SH PEBs (Figure 1c–f). CasaXPS software was used to investigate the peak deconvolution, in addition to the baseline correction of the XPS peaks, as according to the literature [35]. XPS C1s core line peaks of PAA-SH PEBs appear at 284.8 eV (C−C, C−H), 285.4 eV (C−N), and 286.1 eV ($-{\mathrm{COO}}^{-}$) (Figure 1c). XPS O 1s core line peaks appear at 531.9 eV (C =O), 534.6 eV (O−OH), 536.8 eV (C −O), and 539.0 eV ($O=C-O^{-}$) (Figure 1d). In addition, XPS N 1s core line peaks appear at 398.7 eV (N−H) and 400.7 eV (N−C) (Figure 1e). The XPS S 2p core line peak at 162.8 eV (C−SH) was also measured (Figure 1f). These results confirm the connecting of the thiol group and the PAA matrix, as shown in Figure 1a.

**Figure 2:**
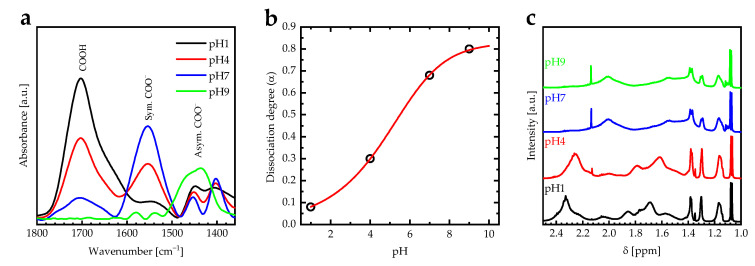
(**a**) FTIR absorbance spectra for carboxylic groups of PAA PEBs at pH 1, 4, 7, and 9 in the range of 1350–1800 cm^−1^ measured at room temperature. (**b**) The dissociation degree (α) calculated using $\alpha =\left[{\mathrm{COO}}^{-}\right]/\left(\left[{\mathrm{COO}}^{-}\right]+\epsilon \left({\mathrm{COO}}^{-}\right)/\epsilon \left(\mathrm{COOH}\right)\left[\mathrm{COOH}\right]\right)$ of PAA PEBs versus pH value. The red line describes the sigmoidal fitting function for the data (**c**) ^1^H NMR spectra for PAA PEBs at the pH values of 1, 4, 7, and 9 measured at room temperature.

FTIR spectroscopy is one of the main techniques used to investigate the swelling behavior and the dissociation degree of the PAA PEBs due to differences in the IR vibrational bands of the carboxylic acid (COOH) and carboxylate (${\mathrm{COO}}^{-}$) groups [13]. The vibrational bands of the carboxylic acid (COOH) and carboxylate (${\mathrm{COO}}^{-}$) groups of the PAA PEBs for different pH values (one, four, seven, and nine) are in the wavenumber range of 1350–1800 cm^−1^ (Figure 2a). In addition, the dissociation degree is calculated using $\alpha =\left[{\mathrm{COO}}^{-}\right]/\left(\left[{\mathrm{COO}}^{-}\right]+\epsilon \left({\mathrm{COO}}^{-}\right)/\epsilon \left(\mathrm{COOH}\right)\left[\mathrm{COOH}\right]\right)$, where $\left[{\mathrm{COO}}^{-}\right]$ and $\left[\mathrm{COOH}\right]$ are the integrated areas of ${\mathrm{COO}}^{-}$ and COOH vibrational bands. The extinction coefficient ratio of the ${\mathrm{COO}}^{-}$ and COOH groups is ($\epsilon \left({\mathrm{COO}}^{-}\right)/\epsilon \left(\mathrm{COOH}\right)=1.8\pm 0.3$) [36]. At pH = 1, the COOH vibrational band at 1700 cm^−1^ dominates the PAA PEBs, with the ratio between the area under the COOH to ${\mathrm{COO}}^{-}$ vibrational bands being about 5.8. Increasing the pH value to 4 by adding NaOH to the PAA stock solution converts the COOH groups of the upper molecules into symmetric ${\mathrm{COO}}^{-}$ and asymmetric ${\mathrm{COO}}^{-}$ groups at vibrational bands of 1553 and 1430 cm^−1^ due to the dissociation of the COOH, with the ratio between the area under the COOH to ${\mathrm{COO}}^{-}$ vibrational bands being about 1.4. Additionally, increasing the pH value to seven leads to an additional conversion of the COOH groups into symmetric ${\mathrm{COO}}^{-}$ and asymmetric ${\mathrm{COO}}^{-}$ groups, with the ratio between the area under the COOH to ${\mathrm{COO}}^{-}$ vibrational bands being around 0.27. Increasing the pH value further to nine leads to a domination of the asymmetric ${\mathrm{COO}}^{-}$ groups at a vibrational band of 1430 cm^−1^, with the ratio between the area under the COOH to ${\mathrm{COO}}^{-}$ vibrational bands decreasing to 0.01. The domination of the negatively charged ${\mathrm{COO}}^{-}$ groups increases the electrostatic repulsion in the polymer chains and stretch the brushes. The titration curve of PAA PEBs was performed by plotting the dissociation degree versus pH and then fitted to the sigmoid function (Figure 2b) [36]. The $pK_{a}$ value of the PAA PEBs is defined as the pH value at the midpoint of the titration curve. Therefore, the effective ${\mathrm{pK}}_{a}$ of the PAA PEBs is about 5.54, which is accepted by the literature [37,38].

In addition, ^1^H-NMR spectra of PAA PEBs for different pH values are shown in Figure 2c. The spectral signatures at ppm 1.10, 1.0–1.7, and 2.32 ppm taken at pH = 1 represent the ${\mathrm{CH}}_{3}$, ${\mathrm{CH}}_{2}$, and CHCOOH protons, respectively. Increasing the pH from one to nine leads to the shifting of the signal of the CHCOOH protons towards smaller chemical shifts, which results in the transformation of the CHCOOH into ${\mathrm{CHCOO}}^{-}$, which thereby confirms the changes in the chemical structure of the PAA PEBs in the FTIR spectra.

**Figure 3:**
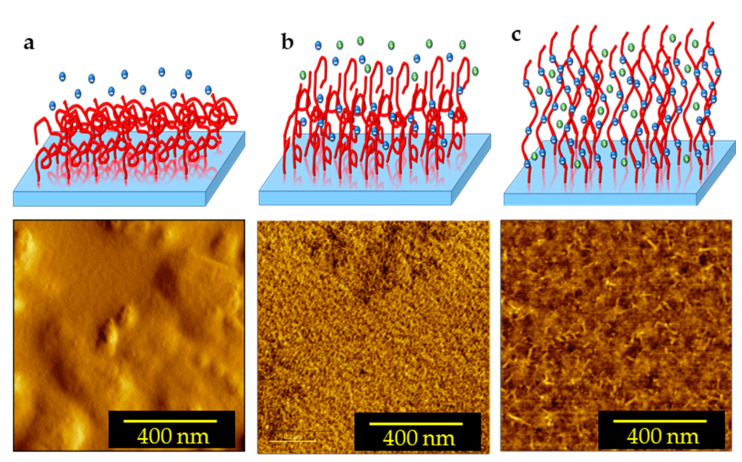
Schematic diagram in addition to 2D AFM images of the switching process of a polymer brush film: (**a**) collapsed brush at pH = 1; (**b**) swollen brush at pH = 7; and (**c**) stretched brush at pH = 9. The negative blue dots: Cl^−^; the positive green dots: Na^+^.

**Figure 4:**
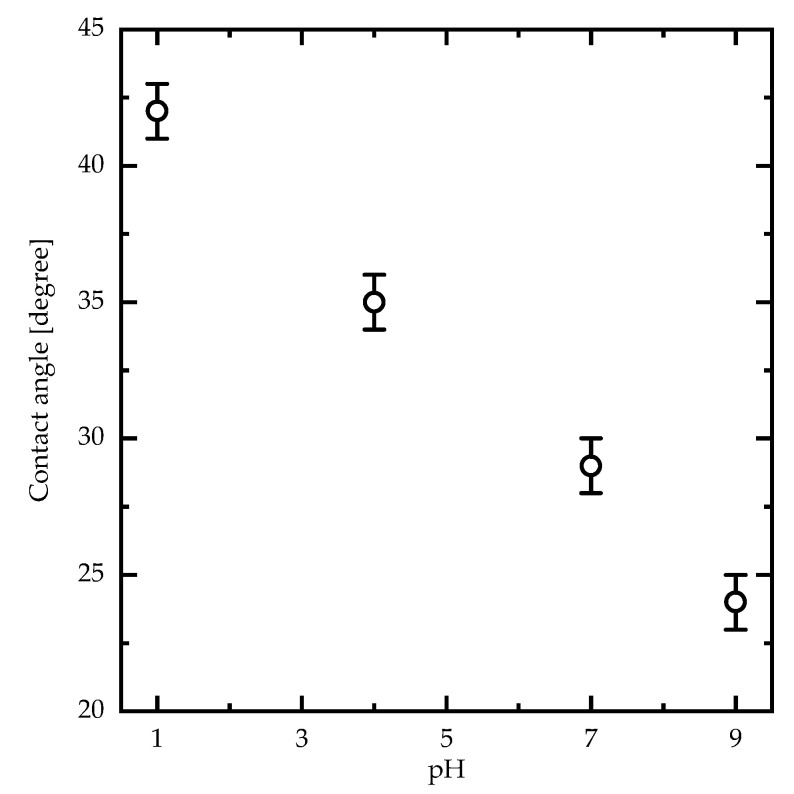
Water contact angle measurements (pH = 7) of PAA PEBs as a function of the pH value measured at room temperature.

To better understand the effects of the dissociation of the carboxylic acid groups of PAA PEBs on the structure following pH variation, schematic diagrams in addition to 2D AFM measurements of PAA PEBs at different pH values (one, seven, and nine) are shown in Figure 3. At pH ~1, the initial state represents the brushes collapsed to the surface (with 1.5 nm roughness), with the domination of the carboxylic groups (COOH) (Figure 3a). This state represents the hydrophobic behavior of the PEBs. The addition of sodium cations through adding NaOH into the preparation solvent until the pH reaches seven leads to dissociation in the COOH groups into ${\mathrm{COO}}^{-}$ groups of the upper molecules of the brushes. Consequently, the collapsed brushes transform into swollen brushes with 0.6 nm roughness (Figure 3b). The cations were unable to reach the deeper molecules of the brush film because of their high density. After pH = 9, the ion concentration is appropriate for reaching all molecules of the PEBs and consequently dissociates most of the COOH groups. This event results in the prevalence of the ${\mathrm{COO}}^{-}$ groups in the brushes (Figure 3c). The domination of the negatively charged ${\mathrm{COO}}^{-}$ groups increases the electrostatic repulsion in the polymer chains and stretches the brushes with 0.3 nm roughness. In this state, the switching process completes, and the brushes swell. Moreover, the PEBs transform from a hydrophobic to a hydrophilic state (Figure 4) [13]. The contact angle of the PAA PEBs at pH = 1 was initially ~42°, which then decreased to ~24° with an increased pH value of nine.

**Figure 5:**
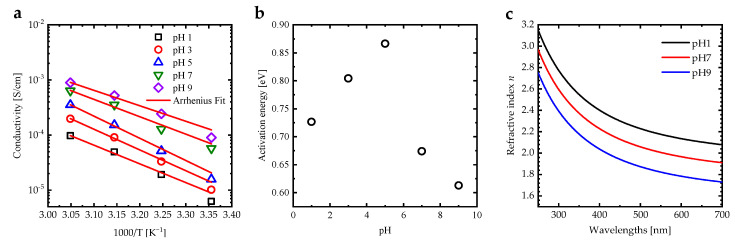
(**a**) Electrical conductivity of the PAA PEBs for different pH values as a function of 1000/T [K^−1^] fitted by the Arrhenius function to calculate the activation energy of the systems, (**b**) activation energy of the PAA PEBs deduced from the Arrhenius function as a function of the pH value, and (**c**) the refractive index spectra of PAA PEBs for the different pH values of 1, 7, and 9.

Increasing the solvent pH increases the electrical conductivity of the PAA PEBs, which can be attributed to the $\mathrm{COOH}\to {\mathrm{COO}}^{-}$ conversion, and from increasing the ion transfer inside the polymer brush layer [13]. In addition, an increase in temperature enhances the electrical conductivity due to a rise in the energy of the segment motion [39]. This results in a concomitant increase in the free volume around the polymer chains as well as the mobility of ions [40]. Figure 5a shows the variation in electrical conductivity (σ) as a function (1000/T(K)) of the PAA PEBs for different pH values. Arrhenius-like behavior for σ can be defined as $\sigma =\sigma_{0}\exp\left(-E_{a}/K_{B}T\right)$, where $\sigma_{0}$ is the pre-exponential factor [41]. The activation energies deduced from σ for the PAA PEBs have an anomalous behavior at pH = 5 (Figure 5b), which can be attributed to the fact that the effective $pK_{a}$ of the PAA PEBs is about 5.54. The refractive index spectrum of the PAA PEBs at pH = 1 exhibits values between 3.15 and 2.08 in the range of 250–700 nm, with a value of 2.09 at a wavelength of 685 nm (Figure 5c). Upon the increase in the pH value to seven, which is the value of the analyte in the SPR measurement, the refractive index decreases to 1.92 at 685 nm. An additional increase in the pH value leads to a decrease in the refractive index to 1.74 at 685 nm. The decrease in the refractive index values upon increasing the pH can be attributed to the change in the PEBs from collapsed to stretched brushes. In addition, the increase in the charge density of the PAA PEBs upon increasing pH values leads to a decrease in the refractive index [42].

**Figure 6:**
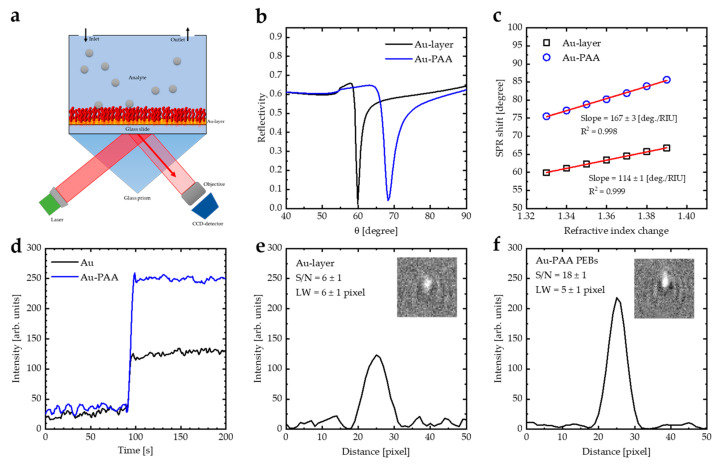
(**a**) Kretschmann’s configuration: a model of a glass prism, a gold layer (41–45 nm), and a PAA layer (30 nm) with an analyte for sensing biomolecules. (**b**) SPR reflectivity curve for Kretschmann’s configuration of the prism–Au-PAA system deduced from WINSPALL software. (**c**) Sensitivity calculations of bare Au and Au-(PAA PEBs) layers from the data calculated by WINSPALL software. (**d**) Time dependence of the detecting event of PSNPs by the Au layer and Au-PAA layers measured by WF-SPRM. (**e**) Line profile plot of PSNPs detected by the Au layer. (**f**) Line profile plot of PSNPs detected by the Au-PAA layers measured by WF-SPRM. Three replicates were performed.

WF-SPRM was employed to record a series of 2D images demonstrating local spatiotemporal changes in intensity, which helps to detect the binding of nano-particles. Thiolated PAA (PAA-SH) was deposited onto the gold sensor surface using the spin coating method (Figure 6a). Figure 6b illustrates the theoretical reflectivity curves of the Au layer and Au-PAA layers, which were modeled via employing WINSPALL software [43]. Coating the Au layer with PAA PEBs increases the incident photon angle and the linewidth (LW). Figure 6c illustrates the shifting in the SPR angle for the Au layer and Au-PAA layers as a function of the refractive index values. The slope of the linear fit represents the sensitivity of the SPR sensor. The SPR sensitivity increases from 114 deg./RIU to 167 deg./RIU as the PAA PEBs appear on the surface of the gold sensing film. Additionally, the figure of merit (FOM) of the sensor, which is defined as the ratio of sensitivity to the LW, was calculated (Table 1) [44]. Coating the Au film with PAA PEBs increases the FOM from 80 to 89 [RIU^−1^].

Figure 6d demonstrates a local intensity of PSNP detected by the Au layer and Au-PAA layers as a function of the measurement time measured by WF-SPRM. The signal-to-noise ratio (S/N) of the Au-PAA layer is higher than the S/N of the Au layer, which is calculated by studying the line profile plot in the x-direction of the images (Figure 6e,f). The S/N for the Au-layer is 6 ± 1, which increased after coating with the PAA layer to 18 ± 1, indicating that the PAA PEBs enhance the sensitivity of the SPR instrument. In addition, the line width (LW) of the recorded image decreased from 6 ± 1 pixels to 5 ± 1 pixels through using the Au-PAA layers, which indicates that the spatial resolution of the recorded image is enhanced. In our previous work [15], we used (PANI-HCl)/Al(NO_3_)_3_ complex composite film as a coating for the Au layer to enhance the sensitivity of the WF-SPRM. We found that the S/N for Au-(PANI-HCl)/Al(NO_3_)_3_ with S/N of 10 ± 1. Therefore, we can conclude that coating the Au layer with PAA brushes is more efficient than our previous work. This can be attributed to the fact that the PAA brushes have a higher refractive index than the (PANI-HCl)/Al(NO_3_)_3_ complex composite film.

## 4. Conclusions

Our work aims to describe the influence of the coating gold sensing surface with the PAA-PEBs on the sensitivity of the WF-SPRM sensor, as well as to provide characteristics of the PAA-PEBs. For this, the pH-dependent switching properties of the PAA-PEBs and their effects on the optical and electrical characterizations were studied. It is demonstrated that, at pH ~1, the initial state of the PEBs corresponds to the brushes that collapse to the surface, with the dominance of the carboxylic groups (COOH). The prevalence of the negatively charged ${\mathrm{COO}}^{-}$ groups increases the electrostatic repulsion in the polymer chains and stretches the brushes. AFM confirms that the PAA PEBs at pH = 1 exhibit collapsed brushes. Increasing the pH to seven leads to the dissociation of the COOH groups of the upper molecules and conversion into the negatively charged ${\mathrm{COO}}^{-}$ groups, and consequently transforms the collapsed brushes into swollen brushes. At pH = 9, the ion concentration is sufficient to reach the deeper molecules of the PEBs that consequently dissociate most of the COOH groups, leading to the prevalence of the ${\mathrm{COO}}^{-}$ groups that, in turn, completes the switching process, leading to stretched brushes. Under increasing pH, the PAA PEBs transform from a hydrophobic to hydrophilic state. Increasing the solvent pH leads to an enhancement in the electrical conductivity of the PAA PEBs. This can be attributed to the $\mathrm{COOH}\to {\mathrm{COO}}^{-}$ conversion. PAA PEBs were deposited on a gold layer using a thiol group (PAA-SH), which was confirmed by XPS and NMR spectroscopy. The signal-to-noise ratio for the Au layer is six, increasing after coating by PAA PEBs to eighteen. In addition, the linewidth of the recorded image decreased from six pixels to five pixels through using the Au-PAA layers. Finally, it was demonstrated that coating the Au layer with PAA PEBs enhances the sensitivity of the SPR sensor and improves the spatial resolution of the recorded image.

## Figures and Tables

**Table 1:** Essential parameters analysis of SPR for bare Au layer and Au-PAA layers.

Layers	$R_{\min}$	$\theta_{\mathrm{SPR}}$ [deg.]	LW [deg.]	S [deg./RIU]	FOM [RIU^−1^]
Equation	--	--	--	S=Δθ/Δn	FOM=S/LW
Au	0.02	59.8	3.8	114	80
Au-PAA	0.05	75.5	4.8	167	89

## Data Availability

Not applicable.
